# Effect of enhanced voluntary physical exercise on brain levels of monoamines in Huntington disease mice

**DOI:** 10.1371/currents.RRN1281

**Published:** 2011-11-08

**Authors:** Thibault Renoir, Caroline Chevarin, Laurence Lanfumey, Anthony J. Hannan

**Affiliations:** ^*^Postdoctoral Research Fellow at Neural Plasticity Group, Florey Neuroscience Institutes, Melbourne Brain Centre, University of Melbourne, Australia; ^†^Research Assistant, addiction and depression Group, Center for Psychiatry and Neuroscience, INSERM U894, University Pierre and Marie Curie, Paris, France; ^‡^Head, addiction and depression Group, Center for Psychiatry and Neuroscience, INSERM U894, University Pierre and Marie Curie, Paris, France. and ^§^Head, Neural Plasticity Group, Florey Neuroscience Institutes, Melbourne Brain Centre, University of Melbourne

## Abstract

Using the R6/1 mouse model of Huntington disease (HD), we have recently shown that voluntary physical activity was able to correct the depressive-like behaviours exhibited by the HD animals at a pre-motor symptomatic stage of the disease. Using the high performance liquid chromatography system, we have now evaluated the effect of exercise on monoamine metabolism in HD mice. We found that serotonin and its metabolite as well as dopamine and noradrenaline were reduced across several brain regions in female R6/1 animals. Our data also suggest that some of these neurochemical deficits were modulated by physical activity, in a genotype-region dependent manner. These newly identified changes could account for some of the behavioural effects of exercise previously reported in HD mice.

## **Introduction**

Huntington disease (HD) is an autosomal dominant neurodegenerative disorder caused by expansion of CAG repeats in exon 1 of the *huntingtin* gene [1]. Clinical onset of HD is determined on the basis of motor symptoms; however the pre-motor stages of the disease are commonly associated with cognitive deficits as well as psychiatric manifestations such as depression [2] [3] [4] [5]. Similar impaired emotionality-related behaviors have been found in transgenic mouse models of HD including R6/1 [6] [7] [8] and YAC128 [9] HD mice.

Serotonin (5-HT) and catecholamines (including noradrenaline (NA) and dopamine (DA)) are known to play a key role in the development of mood disorders [10] [11]. The serotonin-noradrenaline hypothesis of depression is primarily supported by the mechanism of action of the tricyclic antidepressants, which by binding to the serotonin transporter (SERT) and the noradrenaline transporter (NET) result in an increased extracellular concentration of these neurotransmitters. There is also clinical evidence implicating dopaminergic dysfunctions in the pathophysiology of both depression [11] [12] and HD [13]. Interestingly, alterations in 5-HT and/or DA systems have been found in pre-motor symptomatic R6 HD animals [6] [7] [8] [14] [15], and we have recently shown that the associated depressive-like behaviours exhibited by R6/1 female mice were corrected by chronic treatment with the serotonin reuptake inhibitor (SSRI) sertraline as well as voluntary physical activity [7]. 

Many brain disorders (including cognitive and affective abnormalities as well as HD) could potentially benefit from enhanced physical activity. However the effects of exercise on monoamine metabolism have been poorly characterized, especially in motor asymptomatic HD mice. Therefore using the high performance liquid chromatography (HPLC) system, we measured tissue levels of 5-HT, DA and NA in several brain regions (hippocampus, cortex and striatum) of animals (WT and R6/1 HD mice) exposed to running-wheels versus standard housing from 8 to 12 weeks of age.

## **Materials and Methods**

### **Mice**

R6/1 transgenic hemizygote males [16] were originally obtained from the Jackson Laboratory (Bar Harbor, ME, USA) and bred with CBB6 (CBA×C57/B6) F1 females to establish the R6/1 (HD) colony. Animals housed in standard (SH) cages  (15×30×12 cm) were compared with mice housed in large cages (25×37×16 cm) with elevated lids and provided with two running wheels (RW, 12 cm diameter) from 8 to 12 weeks of age. All animals used in this study were group-housed (2 mice from each genotype per cage) and maintained on a 12 h light/dark cycle with access to food and water *ad libitum*. Therefore, we were not able to individually measure the total distance run during the 4 weeks with free access to running wheels. However, we have evidence that female mice used the wheels regardless of genotype (unpublished data using animals grouped by genotype). Furthermore, the cages were monitored regularly and this confirmed that the wheels were frequently used by mice, with either one or two mice observed to run on a single wheel. All experiments were performed on female 12-week mice in accordance with the guidelines of the HFI Animal Ethics Committee and the National Health and Medical Research Council (NHMRC).

### **Whole tissue measurements of serotonin, dopamine, noradrenaline and their metabolites**

Tissue levels of endogenous serotonin (5-HT) and its metabolite 5-hydroxyindolacetic acid (5-HIAA) as well as the catecholamines (dopamine and noradrenaline), were determined as previously published [17]. Dissected brain structures were homogenized in 5-10 volumes (v/w) of ice-cold 0.1 M HClO_4_ containing 1.34 mM disodium EDTA and 0.05% Na_2_S_2_O_5_. Homogenates were centrifuged at 30,000 g for 20 min at 4°C. After neutralization with 2 M KH_2_PO_4_/K_2_HPO_4_, pH 7.4, containing 0.01 mg per mL ascorbate oxidase (Boehringer Mannheim, Meylan, France), supernatants were further centrifuged at 30,000 g for 20 min. Aliquots (10 µL) of clear supernatants were injected into a high performance liquid chromatography (HPLC) column (Ultrasphere IP, Beckman, Gagny, France ; 25x0.46 cm, C18 reversed phase, particle size 5 µm) protected with a Brownlee pre-column (3 cm, 5 µm). The mobile phase for the elution (at a flow rate of 1 mL per min) consisted of (in mM) : KH_2_PO_4_, 70 ; triethylamine, 3.1 ; disodium EDTA, 0.1 ; octane sulphonate, 1.05 ; methanol, 16%, adjusted to pH 3.02 with solide citric acid. The electrochemical detection system (ESA 5011, Bedford, MA , USA) comprises an analytical cell with dual coulometric monitoring electrodes (+50 and +350 mV). The generated signals were integrated by a computing integrator (Millenium 32, Waters, Saint Quentin Fallavier, France). 

### **Statistical analysis**

Statistical analyses were performed using SPSS statistics 17.0 and GraphPad Prism 5.0. Two-way analysis of variance (ANOVAs) were used to examine possible effect of genotype and/or exercise. To determine specific group differences in case of significant main effects (or interaction), the two-way ANOVAs were followed by Fisher’s LSD or Bonferroni post-hoc tests. In all cases, the significance level was set at p<0.05.

## **Results**

**Figure:**
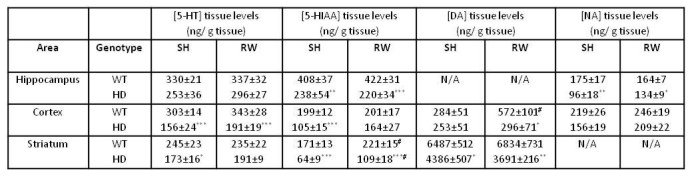

### **Effects of HD mutation and exercise on levels of serotonin and its metabolite**

As shown in figure 1, under standard-housing (SH) conditions the HD mutation produced a decrease of tissue levels of serotonin (5-HT) in the hippocampus (-25%), the cortex (-50%) and the striatum (-30%). However compared to WT controls, two-way ANOVA of hippocampal tissue from HD animals did not reach significance (F_(1,23)_=3.98, p=0.057). Significant reductions were observed in both cortex (F_(1,23)_=45.8, p<0.001) and striatum (F_(1,23)_=8.82, p<0.01) of HD mice. Similar significant deficits of the metabolite 5-HIAA were also displayed by HD animals in all brain regions studied (Figure 1 and Table 1). Finally exercise on running wheel (RW) increased 5-HIAA striatal levels regardless of genotype (F_(1,23)_=15.1, p<0.001).

**Figure:**
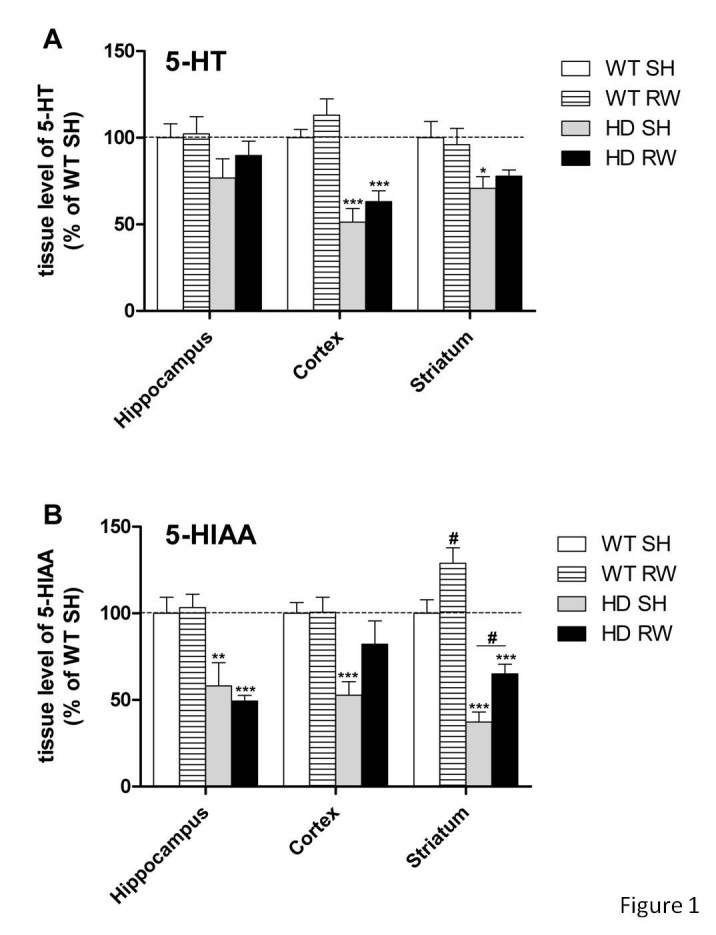
**Figure 1: Effect of HD mutation and voluntary physical exercise on tissue content of 5-HT and 5-HIAA**

Figure 1 illustrates the levels of (A) serotonin (5-HT) and (B) its metabolite (5-HIAA) in various brain regions of WT vs. HD animals housed in standard conditions (SH) or exposed to running wheel (RW). Tissue levels are expressed in percentage (%) of standard-housed WT. Values represent means (± SEM) of independent determinations in 6-8 mice of each genotype. WT vs. HD: (*) p<0.05, (**) p<0.01, (***) p<0.001. SH vs. RW: (#) p<0.05.

### **Effects of HD mutation and exercise on levels of catecholamines**

Tissue content of dopamine (DA) in cortex (F_(1,23)_=4.67, p<0.05) and striatum (F_(1,23)_=24.9, p<0.001) as well as levels of noradrenaline (NA) in hippocampus (F_(1,23)_=14.2, p<0.001) and cortex (F_(1,23)_=4.94, p<0.05) were all decreased in HD mice when compared to WT animals (Table 1). Assessing the effect of running wheel (RW) exercise on brain levels of catecholamines (Figure 2), we found a significant effect of exercise on DA (but not NA) cortical levels in WT mice (F_(1,23)_=6.94, p<0.05). Interestingly, physical activity had no effect on HD animals (F_(1,23)_=1.29, p=0.27).

**Figure:**
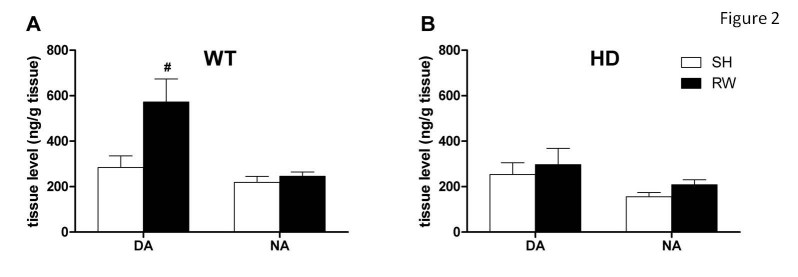
**Figure 2: Effect of HD mutation and voluntary physical exercise on cortical levels of catecholamines** Figure 2 illustrates the levels of dopamine (DA) and noradrenaline (NA) in the cortex of (A) WT vs. (B) HD animals housed in standard conditions (SH) or exposed to running wheel (RW). Tissue levels are expressed in ng/g tissue. Values represent means (± SEM) of independent determinations in 6-8 mice of each genotype. SH vs. RW: (#) p<0.05.

## **Discussion**

As previously shown in R6/2 mice [15], we found that serotonin (5-HT) and its metabolite (5-HIAA) as well as dopamine (DA) and noradrenaline (NA) were reduced across several brain regions in female R6/1 animals. This is the first report of such dopaminergic alterations at the pre-motor symptomatic stage of disease progression. Indeed using a similar number of animals per groups (n=6-8), Reynolds et al. (1999) found reduced DA levels in striatum of R6/2 mice only from 12 weeks of age, paralleling motor impairment and decreased D1/D2 receptor expression [18] observed in these transgenic mice at this stage. Previous studies on R6/1 animals failed to show any effect of the HD mutation on striatal DA tissue content [19] [20], however these contradictory observations were more likely due to statistical limitations (they used n=3 animals per group only) since they nevertheless revealed a strong trend toward a reduced DA levels in HD mice.

Interestingly we found that DA was decreased in the striatum but not in the cortex of R6/1 animals, as recently suggested by another study on R6/2 mice using higher numbers of animals per group (n=15-18) [14]. Altogether our observations are consistent with reduced monoamine levels in the cerebrospinal fluid (CSF) collected from depressed patients [21] (but also see [22]) and could potentially be involved in the development of the depressive-like behaviors we recently reported in pre-symptomatic female R6/1 mice [6] [7] [8].

The beneficial effects of physical activity in alleviating depressive mood are well known and exercise has been often suggested as a form of therapy for depressed patients [23] [24] [25]. Unfortunately similar clinical studies on HD patients (especially at early stage of the disease) have not been undertaken, yet these are crucial to test the relevance of physical activity as a potential therapeutic tool. Promisingly, together with the initiation of the first pre-manifest HD clinical studies [26] [27], recent human data suggest that avoiding a passive lifestyle may significantly delay the onset of HD [[28]. Exercise has been studied in several rodent models of depression [29] [30] [31] [32]. We also have shown than locomotor impairments as well as cognitive and affective deficits displayed by R6/1 animals were ameliorated by wheel running [7] [33] [34], and similar observations have been reported using the R6/2 mice [35] (but also see [36]). Furthermore, environmental enrichment, which also enhances physical activity, has been shown to delay onset of affective, cognitive and motor deficits in R6/1 HD mice [[6] [37] [38]] .  Interestingly, our present HPLC data suggest the turnover of serotonin in the striatum as a possible process to establishing antidepressant effects at a behavioural level since wheel-running resulted in higher concentrations (~70% increase in HD mice) of 5-HIAA in the striatum. The striatum has traditionally been associated with the specific cognitive and motor symptoms of HD; it would be interesting to study the role of striatal pathology in HD symptomatology including affective-associated disorders. Surprisingly, exercise did not correct the striatal DA deficit of HD mice. We also report that cortical DA levels were unchanged in female R6/1 mice after wheel-running, although this same measure was enhanced in WT animals. Finally, NA levels did not seem to be affected by physical activity.

Altogether we show that tissue levels of 5-HT, DA and NA were all reduced in R6/1 mice, even at a pre-motor symptomatic stage of the disease. Our findings also suggest that some of these neurochemical deficits were modulated by physical activity, in a genotype-region dependent manner. However, whether these region-specific identified changes could account for the antidepressant-like effects of exercise (as well as the amelioration of the cognitive deficits and the delayed onset of locomotor symptoms observed in HD mice with access to running wheels) remain unclear. Importantly, experimental prerequisites (e.g. initiating interventions at clear pre-motor stages and designing investigations so as to optimize statistical power) need to be carefully evaluated before undertaking such studies.

## **Acknowledgments**

Leah Leang, Michelle Zajac and Terence Pang for their valuable assistance.

## **Funding information**

This work was funded by NHMRC Project Grants and ARC Future Fellowship (AJH), NHMRC-INSERM Exchange Fellowship (TR).

The funders had no role in study design, data collection and analysis, decision to publish, or preparation of the manuscript.

## **Competing interests**

The authors do not have any conflicts of interests to disclose.

**Correspondence**

 To Thibault Renoir, Behavioral Neurosciences, Florey Neuroscience Institutes, University of Melbourne, Parkville, VIC 3010, Australia. E-mail: tibo.renoir@gmail.com, thibault.renoir@florey.edu.au 
